# RETRACTION: Reliability of Evidence Supporting the Role of Electrical Stimulation in the Treatment of Pressure Ulcers

**DOI:** 10.1111/iwj.70629

**Published:** 2025-04-21

## Abstract

**RETRACTION:** QinX. , ShenH. , ChenR. , YeR. , and HuC. , “Reliability of Evidence Supporting the Role of Electrical Stimulation in the Treatment of Pressure Ulcers,” International Wound Journal 21, no. 2 (2024): e14620, 10.1111/iwj.14620.PMC1083043440261217

The above article, published online on 31 January 2024, in Wiley Online Library (http://onlinelibrary.wiley.com/), has been retracted by agreement between the journal Editor in Chief, Professor Keith Harding; and John Wiley & Sons Ltd. Following an investigation by the publisher, all parties have concluded that this article was accepted solely on the basis of a compromised peer review process. The editors have therefore decided to retract the article. The authors did not respond to our notice regarding the retraction.

**RETRACTION:** X. Qin , H. Shen , R. Chen , R. Ye , and C. Hu , “Reliability of Evidence Supporting the Role of Electrical Stimulation in the Treatment of Pressure Ulcers,” International Wound Journal 21, no. 2 (2024): e14620, 10.1111/iwj.14620.PMC1083043440261217

The above article, published online on 31 January 2024, in Wiley Online Library (http://onlinelibrary.wiley.com/), has been retracted by agreement between the journal Editor in Chief, Professor Keith Harding; and John Wiley & Sons Ltd. Following an investigation by the publisher, all parties have concluded that this article was accepted solely on the basis of a compromised peer review process. The editors have therefore decided to retract the article. The authors did not respond to our notice regarding the retraction.

